# Rates of Viral Evolution Are Linked to Host Geography in Bat Rabies

**DOI:** 10.1371/journal.ppat.1002720

**Published:** 2012-05-17

**Authors:** Daniel G. Streicker, Philippe Lemey, Andres Velasco-Villa, Charles E. Rupprecht

**Affiliations:** 1 Odum School of Ecology, University of Georgia, Athens, Georgia, United States of America; 2 Rabies Program, Centers for Disease Control and Prevention, Atlanta, Georgia, United States of America; 3 Department of Microbiology and Immunology, Katholieke Universiteit Leuven, Leuven, Belgium; Vanderbilt University, United States of America

## Abstract

Rates of evolution span orders of magnitude among RNA viruses with important implications for viral transmission and emergence. Although the tempo of viral evolution is often ascribed to viral features such as mutation rates and transmission mode, these factors alone cannot explain variation among closely related viruses, where host biology might operate more strongly on viral evolution. Here, we analyzed sequence data from hundreds of rabies viruses collected from bats throughout the Americas to describe dramatic variation in the speed of rabies virus evolution when circulating in ecologically distinct reservoir species. Integration of ecological and genetic data through a comparative Bayesian analysis revealed that viral evolutionary rates were labile following historical jumps between bat species and nearly four times faster in tropical and subtropical bats compared to temperate species. The association between geography and viral evolution could not be explained by host metabolism, phylogeny or variable selection pressures, and instead appeared to be a consequence of reduced seasonality in bat activity and virus transmission associated with climate. Our results demonstrate a key role for host ecology in shaping the tempo of evolution in multi-host viruses and highlight the power of comparative phylogenetic methods to identify the host and environmental features that influence transmission dynamics.

## Author Summary

Rapid evolution of RNA viruses is intimately linked to their success in overcoming the defenses of their hosts. Several studies have shown that rates of viral evolution can vary dramatically among distantly related viral families. Variability in the speed of evolution among closely related viruses has received less attention, but could be an important determinant of the geographic or host species origins of viral emergence if certain species or regions promote especially rapid evolution. Here, using a dataset of rabies virus sequences collected from bat species throughout the Americas, we test the role of inter-specific differences in reservoir host biology on the tempo of viral evolution. We show the annual rate of molecular evolution to be a malleable trait of viruses that is accelerated in subtropical and tropical bats compared to temperate species. The association between geography and the speed of evolution appears to reflect differences in the seasonality of rabies virus transmission in different climatic zones. Our results illustrate that the viral mechanisms that are commonly invoked to explain heterogeneous rates of evolution among viral families may be insufficient to explain evolution in multi-host viruses and indicate a role for host biology in shaping the speed of viral evolution.

## Introduction

RNA viruses display exceptionally variable rates of molecular evolution, with up to 6 orders of magnitude in nucleotide substitution rates observed among viral species [1]. Because of the importance of genetic and phenotypic evolution for infecting new host species, evading immune responses and obstructing successful pharmaceutical development, understanding the factors that govern the speed of viral evolution is critical for mitigating viral emergence [2]. To date, explanations for evolutionary rate heterogeneity have been predominately virus-oriented. These have focused on features of genomic architecture that determine underlying mutation rates, aspects of the virus life cycle such as latency and transmission mode that can influence the replication rate within hosts and generation times between hosts, and diversification through positive selection [2]–[5]. Less well understood are the determinants of evolutionary rate variation among closely related viruses (e.g., species within genera) or among lineages of the same viral species circulating in different geographic regions or host species [6], [7]. Because viral genomic features and replication mechanisms are minimally variable at such shallow taxonomic levels, aspects of host biology that influence rates of transmission and replication may be more likely to control the tempo of viral evolution. Consistent with this hypothesis, several human viruses (e.g., HTLV, HIV and Chikungunya virus) that exploit multiple modes of transmission or experience variable immunological pressures within-hosts demonstrate accelerated molecular evolution in conditions associated with enhanced transmission and replication [6], [8], [9].

The propensity of many RNA viruses to ‘jump’ between host species presents an intriguing natural experiment to test whether viral evolutionary rates change according to traits of host species that influence viral replication and transmission or remain evolutionarily conserved along the ancestral history of the virus, reflecting intrinsic biological features of viruses [10], [11]. Moreover, knowledge of accelerated viral evolution in certain reservoir hosts might be useful for predicting the geographic or species origins of future host jumps if faster evolution enhances the genetic diversity on which natural selection may operate. Despite these implications for viral emergence and evolution, the relationship between host biology and viral evolution remains largely unexplored. In two recent analyses, influenza A viruses infecting wild and domesticated birds and infectious haematopoietic viruses of wild and farmed fish each showed some intra-specific variation in viral evolutionary rates. However, host species identity failed to explain these differences, perhaps because high rates of transmission between host groups in each system diluted the effects of any single species on virus evolution [7], [12]. Elucidating the influence of host biology on viral evolution therefore requires large datasets of closely related viruses from multiple ecologically distinct host species that are largely capable of independent viral maintenance.

Rabies virus (*Lyssavirus*, Rhaboviridae) is a globally distributed and lethal zoonotic agent that causes more than 50,000 human deaths annually [13]. Although most human rabies is attributed to dog bites in developing countries, rabies virus also naturally infects over 80 bat species from 4 chiropteran families, and bats represent an increasing source of human and domesticated animal rabies in the Americas [14]. The phylogeny of bat rabies virus reveals viral compartmentalization into many largely species-specific transmission cycles, which have arisen from repeated host shifts within the bat community [15], [16]. Coupled with the diverse behavioral and life history strategies of bats, rabies virus therefore provides a unique opportunity to explore the effects of host biology on virus evolution while explicitly accounting for the effects of the ancestral history of the virus on evolutionary rate by using the rabies virus phylogeny as a guide to past host shifts. Moreover, because many American bat species and genera are broadly distributed with distinct viral lineages in different parts of their geographic range, ecological effects that reflect geographic variation in host behavior can be distinguished from taxonomic effects that arise from the physiological similarity of closely related host species.

Bats represent an especially pertinent taxonomic group for exploring the effects of host biology on viral evolution because of growing interest in how bat ecology influences zoonotic agents such as SARS virus, Nipah virus and Ebola virus [17]. If the behavioral and ecological traits of bats that are hypothesized to influence the maintenance and emergence of pathogens affect either virus replication within hosts or the rate of transmission between hosts, they might also have consequences for viral evolution. For example, overwintering of temperate bats through hibernation or extended bouts of torpor might cause a seasonal pause in transmission and/or decelerated disease progression within hosts, perhaps due to metabolic down regulation of cellular processes or reduced contact rates while bats are inactive [18], [19]. These climate-mediated mechanisms might slow evolution in viruses associated with temperate bats compared to tropical species, where year-round food availability and milder temperatures extend bat and virus activity through all seasons. Next, high contact rates in colonial bats may promote infections with greater virulence and reduced incubation periods, increasing the number of viral generations per unit time and speeding viral evolution [19], [20]. Finally, long distance migration, a relatively common strategy in bats, may slow viral evolution by homogenizing viral populations or by reducing transmission if the physiological stress from migration removes infected hosts from the population, i.e., ‘migratory culling’ [21].

Here, we compile large datasets of bat rabies virus sequences to quantify variation in the evolutionary rate of rabies virus when associated with ecologically and behaviorally distinct reservoir species found in different geographic regions of the Americas. Further, we test whether the tempo of evolution undergoes episodic shifts when rabies virus establishes in new species, implicating host biology as a key driver of viral evolution, or evolves gradually along the ancestral history of the virus, reflecting the greater importance of conserved viral features in controlling evolutionary rates. Finally, we integrate ecological and genetic data through newly developed Bayesian hierarchical phylogenetic models to identify the traits of hosts and the environment that influence rates of viral evolution.

## Results

### Variation in substitution rates across bat rabies lineages

**Figure 1:**
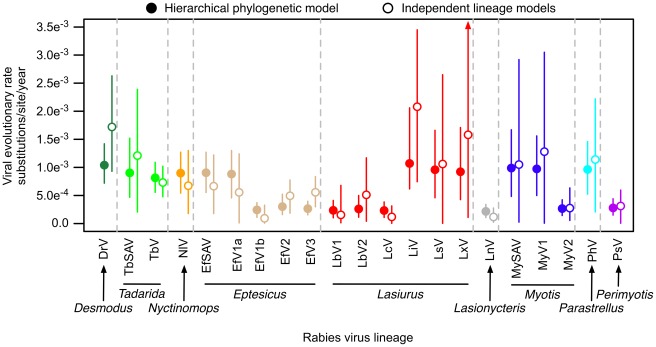
Heterogeneity in evolutionary rates of host-associated bat rabies viruses. Median substitutions per site per year in the 3^rd^ codon position of the nucleoprotein gene for estimates generated by the HPM (filled circles) and ILMs (open circles). Colors and dashed gray lines distinguish bat genera as indicated below the x-axis. Credible intervals denote the 95% highest posterior density interval on evolutionary rate.

We employed maximum likelihood (ML) and Bayesian phylogenetic analyses to define 21 subspecies, species or genus specific lineages of rabies virus for comparative analyses of evolutionary rates (see Materials and Methods for analytical details and operational definitions of lineages). A relaxed molecular clock analysis indicated that viral lineages were relatively young, ranging in age from 83–305 years, with a most recent common ancestor of all bat lineages dating back to 1585 (95% Highest Posterior Density, HPD: 1493–1663; Table S1). To describe the evolutionary rate variation among viral lineages, we focused on substitution rates in the third codon position (CP_3_), as these predominately synonymous substitutions can indicate more clearly how viruses respond to processes affecting their replication rate and generation time between infections [22]. Average substitution rates estimated for each lineage independently (Independent Lineage Models, ILM) and by a hierarchical phylogenetic model (HPM) each spanned approximately one order of magnitude among viral lineages compartmentalized to different host species (ILM range: 8.31×10^−5^–2.08×10^−3^; HPM range: 2.16×10^−4^–1.07×10^−3^ substitutions/site/year). This indicates that select lineages exhibit approximately 5–22 fold acceleration of evolution relative to the slowest evolving viruses. The HPM substantially improved the precision of parameter estimates relative to the ILMs, with only negligible differences in point estimates for most lineages, as previously described in other host-virus systems [8], [23] (Figure 1). Notably, the more extreme values estimated by the ILMs, typically from viral lineages with less informative datasets, were drawn closer to the population mean, suggesting less susceptibility of the HPM to stochastic noise introduced by sampling error and potentially more accurate estimates.

### Virus evolutionary rates are labile following host shifts

**Figure 2:**
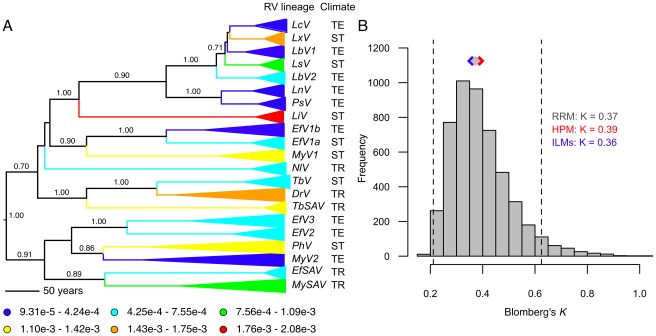
Evolutionary lability of viral substitution rates in the rabies virus phylogeny. (A) Bayesian phylogenetic tree of bat rabies viruses with viral lineage names and climatic regions denoted in black (TE: temperate; ST: subtropical; TR: tropical). Host-associated lineages are condensed to triangles connecting the most recent common ancestor to the sampled branches. Lineages are colored along a blue (slowest) to red (fastest) continuum according to evolutionary rate in CP_3_ using estimates from the Independent Lineage Models (ILMs). Bayesian posterior support values (>0.70) are given above branches to the lineage level only. All colored lineages received Bayesian posterior support values of ≥0.91. (B) Frequency histogram of expected values of Blomberg's *K* from 5000 random assignments of substitution rates estimated from the ILM to lineages. Dashed lines indicate 95% bounds of the null distribution and diamonds denote the median values of *K* for the randomized rate model, RRM (gray), the ILMs (blue), and the HPM (red).

If evolutionary rate is a relatively static trait of viruses, it should be conserved in novel environments and would be expected to reflect the ancestral history of the virus, with closely related viral lineages having similar rates, regardless of their contemporary host environment. We tested the degree of phylogenetic signal in the evolutionary rates of bat rabies virus lineages by quantifying values of Blomberg's *K* (a common statistic for diagnosing phylogenetic non-independence in comparative analysis) [24], [25]. In the context of viral host shifts, significant values of K would indicate that the evolutionary rate tended to remain similar after establishment in the recipient species, whereas weaker values of K would indicate greater shifts in evolutionary rates than expected between the donor and recipient host species. We detected very low and non-significant values of *K* for both rates estimated under the ILMs (*K* = 0.36, *P* = 0.58) and the HPM (*K* = 0.39, *P* = 0.40) and these estimates of *K* did not differ significantly from expected values given our phylogenetic tree and the observed rates under a null model with randomly distributed rates (Figure 2B). Indeed, lineages that shared a most recent common ancestor sometimes had disparate rates of evolution (e.g., LcV and LxV and PhV and MyV2 in Figure 2A), although other closely related virus pairs showed minimal differences in evolutionary rate (e.g., LnV and PsV). Notably, viral lineages maintained by bat species in the temperate zone frequently had slower rates of evolution than lineages from tropical or subtropical bats (Figure 2A). The plasticity of evolutionary rate was corroborated by our Bayesian phylogenetic analysis, which, accounting for uncertainty in the evolutionary history of bat rabies lineages, found no correlation in the evolutionary rates along consecutive branches in the bat rabies virus phylogeny (covariance = 0.005, 95% HPD: −0.051–0.058). These analyses demonstrated that rates of viral evolution may be altered or conserved following establishment in new host species and point to host biology rather than the ancestral history of the virus as the most likely candidate for controlling rabies virus evolution. Using a phylogeny of bat hosts from mitochondrial sequence data, we found that viral evolutionary rates were similarly unconstrained by host evolutionary relatedness (ILMs: *K* = 0.07, *P* = 0.18; HPM: *K* = 0.21, *P* = 0.08), such that viruses associated with closely related bat species or sub-species often had dissimilar evolutionary rates (Figure 1, Figure S1).

### Local host environment determines virus evolutionary rates

Because the evolutionary rates of rabies virus lineages could not be explained by the reservoir host or virus phylogeny alone, we tested whether physiological, ecological or environmental traits of hosts (Table S2) could instead determine viral evolutionary rates using a generalized linear model (GLM) comparison approach. The factors that we tested included both evolutionary conserved traits of bats (i.e., basal metabolic rate, coloniality, long-distance migration) and descriptors of sampling effort and climatic region of viral lineages that were largely independent of the bat phylogeny (Table S3). The GLM identified the climatic region of bat taxa as the single strongly supported predictor of viral evolution (Akaike importance weight = 1.0), alone explaining 66% of the variance in viral evolutionary rates (*F*_1,19_ = 37.2, *R^2^* = 0.66, P<0.0001; Table S4). A phylogenetic generalized least squares regression approach, designed to control for any residual effects of the virus phylogeny on rates of viral evolution, yielded similar results (Table S5).

**Figure 3:**
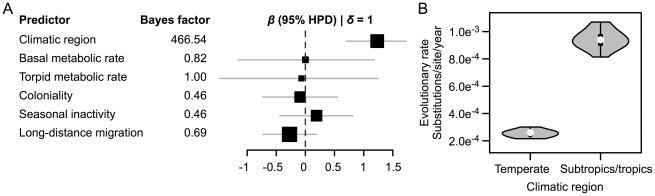
Predictors of viral evolutionary rate from the Bayesian hierarchical phylogenetic model. (A) Effect sizes (*β*) on a log scale for each predictor were conditioned on the inclusion of that term in the model (i.e., *β* | *δ* = 1). Climatic region, coloniality, seasonal inactivity and long distance migration were categorical variables. Horizontal lines are the 95% highest posterior density intervals on conditional effect sizes and squares (median effect sizes) are proportional to effect sizes. (B) Violin plot showing the effect of climatic region on viral evolutionary rate. White points, black boxes and whiskers indicate the median, inter-quartile range and the total range of values for that group, respectively. The gray shading shows the probability density of evolutionary rate at different values.

A weakness of the statistical methods described above was that they could not account for the often-substantial uncertainty in point estimates of evolutionary rate from the ILMs (Figure 1). We therefore incorporated the same categorical and continuous terms directly into a Bayesian HPM that allowed us to simultaneously quantify the posterior distribution of the rate of evolution for each viral lineage from the molecular sequence data, estimate regression model parameters and compare candidate models using Bayes factors (BF) while accounting for phylogenetic uncertainty. The Bayesian model echoed the strong support for accelerated viral evolution in the tropics and subtropics relative to viruses restricted to the temperate zone (log effect size, *β* = 1.24 [95% highest posterior density = 0.72–1.76]; BF = 466.54) with negligible support for all other predictors (BF≤1; Figure 3A). On average, rabies viruses found in tropical or subtropical bat species accumulated 9.44×10^−4^ (ILM: 1.15×10^−3^) substitutions per site per year (subs/site/year), while viruses in temperate bats accumulated only 2.53×10^−4^ (ILM: 2.92×10^−4^) subs/site/year (Figure 3B) – a nearly fourfold deceleration of viral evolution in temperate bats.

## Discussion

By applying an integrated ecological and genetic comparative approach to a unique dataset spanning hundreds of viruses isolated from many host species, our study demonstrated strong effects of host biology on the tempo of molecular evolution in an RNA virus. We observed approximately an order of magnitude of variation in rates of evolution among rabies viruses, indicating that certain lineages evolve up to 22 times faster than others, depending on the reservoir species (Figure 1). Such rate heterogeneity within a single virus species is exceptional given that similar variation is more commonly observed among different viral families and comparable to the variation in mitochondrial DNA divergence rates between vertebrate taxa genetically isolated for millions of years (e.g., whales versus rodents) [26]. Moreover, the young age of viral lineages in our analysis indicated that evolutionary rate could be altered within decades, consistent with rapid rate adjustment after shifts to new host species (Table S1). Since the genomic structure, transmission route and replication mechanisms are not known to vary among rabies virus lineages, the plasticity in evolutionary rate that we observed could only have arisen from ecological differences among reservoir bat species that influence transmission and/or replication.

Because the tempo of evolution shifted freely through the ancestral history of rabies virus, we sought to identify the traits of bat species that influenced viral evolution. Strikingly, the viral molecular clock ticked nearly four times slower in rabies viruses in temperate bat species compared to tropical and subtropical species (Figure 2A, Figure 3B). This pattern could not be explained by geographic structuring of bat diversity or evolutionary conserved aspects of bat physiology or behavior because several widely distributed bat species and genera supported disparate rates of evolution in viral lineages circulating in different climatic regions (Figure 1). This resulted in a weak phylogenetic signal of viral evolution in the bat phylogeny (Figure S1). Similarly, the geographic clustering of viral evolutionary rates in our analysis was unlikely to reflect contrasting patterns of natural selection among climatic regions because evolutionary rates were estimated exclusively from the third codon position of sequences, where most nucleotide substitutions are synonymous, and therefore most likely to be neutral.

Alternative explanations for the relationship between climatic region and the rate of viral evolution parallel previous work on latitudinal gradients of molecular evolution in free-living plants and animals. This work has suggested that accelerated evolution in the tropics might arise from shorter generation times and higher metabolic rates (potentially increasing mutations through greater production of free radicals) associated with warmer environmental temperatures [26], [27]. The latter effect of temperature on host metabolism is unlikely to influence the evolutionary rates of viruses found in heterothermic species such as bats, which also lack a strong relationship between latitude and basal metabolic rate [28]. In our study, the independence of the evolutionary rate of rabies virus from bat metabolic rates further argued against a metabolism-mediated relationship between environmental temperature and viral replication (Figure 3). In contrast, generation times between viral infections likely differed among climatic regions in ways that could produce the patterns of viral evolution that we observed. Specifically, year-round transmission and replication may increase the annual number of viral generations in tropical and subtropical bats relative to seasonal pulses of transmission in temperate species, thereby speeding evolution. Indeed, when we conditioned our Bayesian analysis on 231 iterations of the HPM that lacked the climatic region term, seasonal inactivity was the only predictor that gained strong statistical support (BF = 36) with significantly faster viral evolution in bat species that remain active year-round relative to species that hibernate or use prolonged torpor during winter (*β* = 1.01 [95% highest posterior density = 0.39–1.52]). In our statistical models, the selection of climatic region (a surrogate of seasonal activity) rather than records of activity collected from the literature may be explained by poor understanding of the occurrence and duration of seasonal inactivity and torpor for many bat species [29]. Because assignment of overwintering records often required generalization of a few observations to an entire species range, climatic region may have been a more accurate descriptor of seasonal activity, especially for species that demonstrate geographically variable overwintering behaviors [30]. Still, the effects of variable transmission dynamics on viral evolution that we suggest should be confirmed in other host-virus systems with natural variation in seasonality or through experimental manipulation of virus transmission.

Rapid evolution can enable the cross-species emergence of RNA viruses by increasing the genetic and phenotypic variation available to natural selection [2]. However, whether accelerated viral evolution increases the likelihood of emergence depends on the underlying forces of selection in the reservoir host, whether faster evolution increases variability in the genomic regions that are key to adaptation, and the strength of other ecological and physiological barriers to infecting new host species. In the case of bat rabies, faster viral evolution seemed to arise through an epidemiological mechanism: a greater number of viral generations per year. Enhanced transmission could therefore increase the likelihood of viral emergence in the tropics/subtropics by allowing more ecological opportunities for cross-species transmission. However, whether escaping seasonal transmission bottlenecks also provides an *evolutionary* advantage for host shifting requires understanding how the evolutionary rates that we estimated relate to standing diversity in the genomic regions that mediate viral adaptation to new host species. Although previous work in plant RNA viruses has demonstrated effects of host species on viral genetic diversity, the role of evolutionary rate in generating these effects and their impacts on cross-species emergence remain unknown [31]. Therefore, identifying the genomic regions that enable rabies virus host shifts and the ecological and evolutionary factors that may contribute to their diversity should be a key goal for predicting future rabies virus emergence.

Beyond bat rabies, accelerated molecular evolution in tropical environments is a topic of general interest for understanding the maintenance and emergence of viral infections that occur across geographic regions or experience altered transmission dynamics as a result of anthropogenic environmental change. For example, lineages of Chikungunya virus evolve more slowly in seasonal African environments where mosquito populations and transmission dynamics are more variable relative to urban transmission cycles in Asia, where consistently large human and mosquito populations may shorten times between infections and support epidemic maintenance over multiple years [9]. Similarly, viruses such as influenza show reduced seasonality in the tropics relative to temperate zones [32]. Our results would predict that this sustained transmission might accelerate evolution in tropical viral lineages relative to their temperate counterparts if each is maintained independently. We therefore emphasize the need to consider not only functional traits of viruses, but also the seasonality and epidemiological dynamics of the host-virus interaction for a more complete understanding of the tempo of viral evolution.

In conclusion, our study demonstrated a relationship between climate and the speed of viral evolution, which reinforces similar geographic structuring of molecular evolution as observed in free-living plants and animals [33]. This speeding up of evolution appeared to be driven by changes in the generation time between infected hosts, but not host genetic relatedness, indirect effects of temperature on host metabolism or differences in selective pressures. The broad geographic and host range of many rapidly evolving viruses, together with the increasing availability of molecular sequence data, makes them an ideal, real-time system to examine the epidemiology and evolution of host-pathogen ensembles. Our study revealed the complex interplay between ecological and evolutionary dynamics in multi-host viruses and highlighted an equally integrated framework for dissecting those interactions by combining ecological and genetic data.

## Materials and Methods

### RT-PCR amplification and sequencing of rabies viruses

Viral sequences were generated from tissue samples from naturally infected bats that were collected by state public health laboratories following human or domesticated animal exposures. Total RNA was extracted directly from bat brains without passage using Trizol (Invitrogen, Carlsbad, CA) according to the manufacturer's instructions. A 903 bp fragment comprising the last 687 bp of the N gene, a non-coding region following the *3′* end of N and a small fragment of the phosphoprotein gene was amplified by reverse transcription-polymerase chain reaction and sequenced using oligonucleotide primers 550F and 304R, as described previously [16]. To enable comparison with existing sequences in GenBank, only the coding region of N was used in subsequent analyses. Sequences generated herein have been deposited into GenBank under accession numbers JN594500–JN594503 and DQ445318–DQ445330, DQ445352 (updated sequences). An additional 650 complete or partial rabies virus nucleoprotein gene sequences that were associated with bats from North and South America and contained information on sampling date to year were downloaded from GenBank.

### Biological and ecological data for bat host species

We collected information on the overwintering activity patterns, migratory behavior, roosting behavior and metabolic rates (basal and during seasonal torpor) of the bat species that served as reservoir hosts for the rabies viruses included here from the primary literature and existing databases (Table S2). Long distance migration was defined as seasonal movement of individual bats of at least 1000 km [34]. For 4 species for which basal metabolic rate (BMR) data were unavailable, we borrowed values from species within the same genus or family that had similar body mass. Notably, body mass explains >92% of variation in BMR in bats and phylogeny explains much of the residual variation [29]. When torpid metabolic rate (TMR) estimates spanned a range of temperatures or spatial locations, rates were selected to match the conditions that bats are likely to experience in northern latitudes of their range where hibernation/torpor is most important. To calculate TMR for species that lacked values in the literature, we estimated the relationship between BMR and TMR for the 9 species in our dataset for which both values were available. This relationship was remarkably consistent across species (TMR = 2.2–3.2% of BMR, mean = 2.8%), with the exception of *Tadarida brasiliensis*, for which TMR was 12.6% of BMR. Because the reported estimate of TMR for that species (and for the other subtropical and tropical species in our study) likely represented a daily torpor rather than longer-duration, seasonal torpor, it was excluded from the calculation of the average mentioned above [35]. Overwintering activity was challenging to classify because the frequency and duration of bat activity during winter are poorly understood for many temperate bat species and can vary substantially throughout their geographic range [30], [36]. Therefore, we classified species as inactive during winter if extended bouts of seasonal torpor or hibernation were reported in any part of their geographic range, recognizing that this classification may have been overly conservative. As a potentially more geographically sensitive proxy of year-round activity, the climatic region (tropical, subtropical, temperate) of the center of the geographic range of each viral lineage was also recorded. North American lineages circulating between 35° and 23.5° latitude and South American lineages circulating south of −23.5° latitude that support mean winter temperatures of ≥10°C were considered subtropical, and lineages found towards and away from the equator relative to these latitudes were classified as tropical and temperate, respectively [37].

### Selection of lineages for hypothesis testing

To define phylogenetic lineages to be included in subsequent analyses, ML and Bayesian phylogenetic analyses were performed using Garli v.0.96b and BEAST v.1.6.1, respectively [38], [39]. The ML analysis used the General Time Reversible (GTR) model of nucleotide substitution with invariant sites (I) and Γ distributed rate variation among sites as suggested by Akaike's information criterion corrected for small samples size (AICc) in jModeltest [40]. The ML tree was estimated by 5 independent searches with random starting trees, followed by 5 additional searches using the best tree from the previous set of searches as the starting tree. For the Bayesian analysis, we linked substitution rates for the first and second codon positions (CP_12_) and allowed independent rates in CP_3_. Separate substitution models were selected for CP_12_ and CP_3_ in jModeltest using AICc after partitioning aligned sequences by codon position. The BEAST analysis therefore applied the TIM1ef+I+Γ substitution model to CP_12_ and the TVM+Γ substitution model to CP_3_. We used the Bayesian skyride model as a flexible demographic prior for viral effective population size and an uncorrelated lognormal relaxed molecular clock to accommodate rate variation among lineages. Five independent Markov Chain Monte Carlo (MCMC) analyses were run for 50 million generations each, with samples from the posterior drawn every 50,000 generations following variable burn-in periods based on convergence of likelihood values and model parameters. The results from the five runs were combined to generate a maximum clade credibility tree and divergence time summaries. Lineages for subsequent analyses of substitution rates included those that *(i)* contained at least 8 sequences (mean = 30.9 sequences), *(ii)* were supported by Bayesian posterior probabilities of >0.9 and *(iii)* were sampled over a minimum time span of 4 years (mean = 19.4 years). These conditions aimed to achieve a compromise between precision in estimates and hypothesis testing ability. To ensure that sparsely sampled viral lineages did not bias our central findings, statistical analyses were conducted with covariates designed to identify effects of sampling heterogeneity or using hierarchical phylogenetic modeling to incorporate uncertainty in rate estimates. Of the 28 viral lineages identified in the initial phylogenetic analyses, 21 fit our criteria for inclusion in subsequent analyses, amounting to a final dataset of 648 sequences collected between 1972 and 2009 from 21 bat species or sub-species.

### Independent estimation of nucleotide substitution rates in bat rabies lineages

Sequence alignments were constructed for each viral lineage and nucleotide substitution models were selected for CP_12_ and CP_3_ as described above. For each lineage, the substitution rate was estimated in BEAST assuming an uncorrelated lognormal relaxed molecular clock to accommodate rate variation along branches and the Bayesian skyline model as a flexible demographic prior that could be applied to all viral lineages. The evolutionary rate in CP_3_ was calculated by multiplying the mean substitution rate by the relative rate parameter for that partition. Each simulation was run for at least 100 million generations, with parameters sampled every 5,000–10,000 generations. The first 10% of each run was discarded prior to the construction of the posterior probability distributions of parameters. Each analysis was run sufficiently long that effective sample sizes for parameters were >200 and results of several independent runs were combined. Analyses of evolutionary rates focused on substitution rates in CP_3_ since these largely synonymous substitutions reflect differences in evolution associated with generation time [22]. However, rates in CP_12_ were closely correlated with CP_3_ rates (*r* = 0.87, *P*<0.0001). Rate estimates for all codon partitions are shown in Table S6 and Table S7.

### Bayesian hierarchical inference of nucleotide substitution rates and hypothesis testing

The separate estimation of substitution rates for each rabies virus lineage assumes complete independence of parameters across viral lineages, but this is unlikely the case given the close evolutionary relationships among lineages and biological similarities of the processes of infection and replication among lineages. Because the quantity of data varied among lineages (the number and temporal range of sequences), independent estimation in sparsely sampled lineages may lack power, causing imprecise estimates. HPMs have been proposed to improve the precision of parameter estimates for partially independent datasets such as these (e.g., populations of HIV within different patients) by assuming that individual lineage parameters vary around a shared unknown, but estimable population mean [23]. More recently, tools have been developed within BEAST to incorporate fixed effects into HPMs and to select among candidate models via Bayes factors [8]. These models take the general form of:
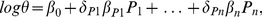
(1)where *θ* is the evolutionary response variable of interest (here, the rate of molecular evolution in CP_3_), *β_0_* is an unknown grand mean, *δ* is a binary indicator that tracks the posterior probability of the inclusion of predictor, *P*, in the model and *β* is the estimated effect size of predictor *P*. The use of binary indicator variables (*δ*) within the MCMC search allows for a Bayesian stochastic search variable selection approach that simultaneously estimates the posterior probabilities of parameters for all possible combinations of predictors and allows for calculation of the Bayes factor support for individual predictors as the ratio of the posterior odds to the prior odds of each predictor in the model.

We constructed a HPM for the 21 bat rabies virus lineages that assigned separate strict molecular clocks to CP_12_ and CP_3_ of each viral lineage and included fixed effect predictors of the evolutionary rate in CP_3_. For each CP, the evolutionary rate parameter of the molecular clock, the parameters of the GTR substitution model and the shape parameter of the discrete Γ distribution were modeled hierarchically across lineages, with all other parameters varying independently across data partitions. Results were robust to simpler substitution models lacking Γ heterogeneity within each codon partition and to statistical analyses using the rate of evolution averaged across all three codon positions. The fixed effects in the full model included climatic region (temperate vs. tropics/subtropics), mass-independent BMR, mass-independent TMR, coloniality (solitary vs. colonial), seasonal activity (non seasonal vs. hibernation/periodic seasonal torpor) and long-distance migration (migrants vs. non-migrants) (Table S2). Climatic region was condensed to two categories in the HPM based on exploratory analyses that demonstrated no difference in evolutionary rate between tropical and subtropical viral lineages. All continuous variables were log transformed. The HPM was implemented in BEAST using four independent MCMC searches of 150 million generations each, with the posterior sampled every 5,000 generations. Results from the four runs were combined after discarding the first 10% of each. Effect sizes of predictors reported from the HPM were calculated conditionally on the portion of the posterior distribution for which the respective effects were included in the model (i.e., *β_i_*|*δ_Effect i_* = 1). Similarly, evolutionary rate estimates from the HPM were calculated conditionally on samples of the posterior in which the statistically supported predictor was included in the model (107,773/108,004 samples). Source files for the BEAST analysis and R scripts for conditional effect size and parameter estimation are available from the corresponding author upon request.

In addition to the Bayesian hierarchical hypothesis testing framework described above, we conducted a more traditional GLM analysis of host predictors of mean viral evolutionary rates and a phylogenetic least squares (PGLS) regression analysis. These analyses contained the factors included in the HPM above, but because these methods do not account for uncertainty in the estimation of evolutionary rates (Figure 1), we also included several factors to identify the influence of estimation error: the number of years spanned and the number of sequences that comprised each dataset. For the GLM analysis, an initial model containing all terms was simplified using an exhaustive search of possible models using AICc in the *glmulti* package of R [41], [42]. Models with Akaike weights within 10% of the highest were retained in the confidence set shown in Table S4. The PGLS regression was conducted in the *caper* package of R, using the Pagel's λ statistic to account for phylogenetic non-independence of viral evolutionary rates [43]. Because λ for climatic zone was low in the virus phylogeny (λ = 4.5e-5), we had little power to reconstruct ancestral climatic states. This precluded testing whether viral jumps between climatic zones were correlated with directional changes in viral evolution.

### Phylogenetic signal in viral substitution rates and host traits

Blomberg's *K* measures the degree of phylogenetic non-independence of species traits, with values ranging from 0 to infinity [24], [25]. Values of *K*<1 indicate less phylogenetic signal (more trait lability) than expected under a Brownian motion model of evolution and *K*>1 indicate more correlation with phylogeny than expected. The *K* statistic was calculated for the ILM and HPM sets of rate estimates using the topology of the rabies virus phylogeny in Figure 2A in the *picante* package of R [42], [44]. The statistical significance of estimates of *K* was tested by comparing the expected distribution of values on our phylogenetic tree to 5,000 randomizations of observed rates along the tips of the tree. A similar analysis using the ML phylogeny of bats estimated from published mitochondrial cytochrome oxidase I sequences assessed whether the ecological and physiological similarity of closely related host species promotes evolution towards similar rates of viral evolution (Table S3, Figure S1).

## Supporting Information

Figure S1 The maximum likelihood phylogenetic tree of bat taxa in the study estimated from mitochondrial DNA sequences (COI) and the corresponding rates of viral evolution for associated rabies virus lineages. The figure shows the tree with the highest log likelihood after five replicate ML searches in Garli using the TIM1+I+Γ model of nucleotide substitution suggested by AICc in jModeltest. A single outgroup sequence from *Emballonura alecto* (Genbank Accession No. HM540244) was pruned from the tree. The scale bar indicates substitutions per site. The rates shown are estimates from the independent lineage models, and neither these rates nor the hierarchical phylogenetic model rates showed significant association with the bat phylogeny. Parentheses to the right of species names contain viral lineage abbreviations as in Table S1.
(DOC)

Table S1 Host species, divergence time summaries and sampling information for rabies virus lineages. Two estimates of divergence times are shown. The first estimate of the time since the most recent common ancestor (“Stem branch TMRCA") includes the stem branch leading to existing lineage diversity. The second estimate of the TMRCA includes only existing viral genetic diversity. The “SAV" notation denotes lineages found in South American bat populations/species that contain a congeneric North American member in the dataset.
(DOC)

Table S2 Ecological traits of bat species and data sources. Mass independent values of basal metabolic rate (BMR) and torpid metabolic rate (TMR) were used. Rates that were calculated using data on body mass and metabolic rates (see Materials and Methods) are indicated by “a". Climatic regions abbreviated as follows: TR = tropical; ST = subtropical; TE = temperate. Representative species trait values are reported for viruses that circulate in several species of *Myotis* in the western United States.
(DOC)

Table S3 Phylogenetic signal of bat and virus traits in the bat phylogeny as measured by Blomberg's *K* and Pagel's *λ*. Values of Blomberg's *K* were estimated for continuous traits with significance tested by 5000 randomizations of trait values on the phylogenetic tree of bats from COI sequences (Figure S1). Maximum likelihood (ML) values of *λ* were estimated using the *Geiger* package of R with significance determined by likelihood ratio tests comparing models assuming the ML estimate of *λ* to models assuming no phylogenetic signal (*λ* = 0) with 1 degree of freedom. When only two categories of climatic zone were included (tropical and subtropical versus temperate) the ML value of *λ* was 1; however, this estimate was only marginally significantly better than the model assuming *λ* = 0 (*P* = 0.06).
(DOC)

Table S4 The confidence set of generalized linear models examined to explain viral evolutionary rate. All models included a significant intercept term and had full model *P* values<0.0001. Abbreviated terms are defined as follows: BMR: mass-independent basal metabolic rate; TMR: mass-independent torpid metabolic rate; n: number of sequences per lineage; nyrs: range of years spanned per lineage. AIC weights (*w*) describe the relative likelihood for each model given the set of models considered.
(DOC)

Table S5 Phylogenetic generalized least squares regression support for the confidence set of models found in the generalized linear model analysis (see Table S4 for explanations of terms). Pagel's λ was estimated using the rabies virus phylogeny (topology from Figure 2A) and observed trait data.
(DOC)

Table S6 Evolutionary rate estimates by codon partition for independent lineage models. Numbers in parentheses are the 95% highest posterior density around the median rate.
(DOC)

Table S7 Evolutionary rate estimates by codon partition from the hierarchical phylogenetic model. Numbers in parentheses are the 95% highest posterior density around the median rate and were calculated conditionally on the portion of the posterior distribution for which the significant effect (climatic region) was included in the model (i.e., *β_i_*|*δ_Effect i_* = 1).
(DOC)
