# Supplementary material for: Rates of Viral Evolution Are Linked to Host Geography in Bat Rabies
Source: PLoS Pathog. 2012 May 17;8(5):e1002720. doi: 10.1371/journal.ppat.1002720 (PMC3355098; doi:10.1371/journal.ppat.1002720)
Supplement: Figure S1 — The maximum likelihood phylogenetic tree of bat taxa in the study estimated from mitochondrial DNA sequences (COI) and the corresponding rates of viral evolution for associated rabies virus lineages. The figure shows the tree with the highest log likelihood after five replicate ML searches in Garli using the TIM1+I+Γ model of nucleotide substitution suggested by AICc in jModeltest. A single outgroup sequence from Emballonura alecto (Genbank Accession No. HM540244) was pruned from the tree. The scale bar indicates substitutions per site. The rates shown are estimates from the independent lineage models, and neither these rates nor the hierarchical phylogenetic model rates showed significant association with the bat phylogeny. Parentheses to the right of species names contain viral lineage abbreviations as in Table S1. (DOC) [file ppat.1002720.s001.doc]

**Figure S1**

Figure S1. The maximum likelihood phylogenetic tree of bat taxa in the study estimated from mitochondrial DNA sequences (COI) and the corresponding rates of viral evolution for associated rabies virus lineages. The figure shows the tree with the highest log likelihood after five replicate ML searches in Garli using the TIM1+I+Γ model of nucleotide substitution suggested by AICc in jModeltest [1,2]. A single outgroup sequence from *Emballonura alecto* (Genbank Accession No. HM540244) was pruned from the tree. The scale bar indicates substitutions per site. The rates shown are estimates from the independent lineage models, and neither these rates nor the hierarchical phylogenetic model rates showed significant association with the bat phylogeny. Parentheses to the right of species names contain viral lineage abbreviations as in Table S1.

**Figure S1 References**

1. Zwickl DJ (2006) Genetic algorithim approaches for the phylogenetic analysis of large biological sequence datasets under the maximum likelihood criterion. The University of Texas at Austin.

2. Posada D (2008) jModelTest: Phylogenetic model averaging. Molecular Biology and Evolution 25: 1253-1256.
