# Supplementary material for: Rates of Viral Evolution Are Linked to Host Geography in Bat Rabies
Source: PLoS Pathog. 2012 May 17;8(5):e1002720. doi: 10.1371/journal.ppat.1002720 (PMC3355098; doi:10.1371/journal.ppat.1002720)
Supplement: Table S1 — Host species, divergence time summaries and sampling information for rabies virus lineages. Two estimates of divergence times are shown. The first estimate of the time since the most recent common ancestor (“Stem branch TMRCA") includes the stem branch leading to existing lineage diversity. The second estimate of the TMRCA includes only existing viral genetic diversity. The “SAV" notation denotes lineages found in South American bat populations/species that contain a congeneric North American member in the dataset. (DOC) [file ppat.1002720.s002.doc]

**Table S1**

| **Lineage** | **Host species** | **Stem branch TMRCA (95% HPD)** | **TMRCA (95% HPD)** | **Date range of sequences** | **Number of sequences** |
| --- | --- | --- | --- | --- | --- |
| DrV | *Desmodus rotundus* | 202 (146-308) | 171 (122-229) | 22 | 60 |
| TbSAV | *Tadarida brasiliensis brasiliensis* | 304 (227-407) | 32 (18-48) | 12 | 10 |
| TbV | *Tadarida brasiliensis mexicana; T. b. cynocephala* | 195 (139-261) | 65 (44-95) | 26 | 81 |
| NlV | *Nyctinomops laticaudatus* | 155 (81-240) | 55 (29-101) | 16 | 17 |
| EfSAV | *Eptesicus furinalis* | 280 (188-356) | 34 (22-49) | 10 | 14 |
| EfV1a | *Eptesicus fuscus bernardinus* | 185 (107-271) | 52 (32-85) | 10 | 20 |
| EfV1b | *Eptesicus fuscus fuscus* | 185 (107-271) | 127 (74-202) | 19 | 26 |
| EfV2 | *Eptesicus fuscus fuscus; E. f. bernardinus* | 243 (171-317) | 80 (54-107) | 22 | 32 |
| EfV3 | *Eptesicus fuscus bernardinus* | 243 (171-317) | 145 (103-204) | 34 | 69 |
| LbV1 | *Lasiurus borealis* | 84 (62-108) | 62 (46-83) | 20 | 17 |
| LbV2 | *Lasiurus borealis* | 103 (70-134) | 44 (34-57) | 25 | 46 |
| LcV | *Lasiurus cinereus* | 87 (64-111) | 47 (37-60) | 28 | 72 |
| LiV | *Lasiurus intermedius floridanus* | 147 (82-224) | 46 (31-69) | 17 | 11 |
| LsV | *Lasiurus seminolus* | 88 (63-111) | 41 (31-66) | 27 | 8 |
| LxV | *Lasiurus xanthinus* | 85 (56-113) | 33 (19-50) | 11 | 9 |
| LnV | *Lasionycteris noctivagans* | 98 (72-129) | 51 (37-77) | 30 | 40 |
| MySAV | *Myotis nigricans* | 281 (193-363) | 161 (95-233) | 4 | 12 |
| MyV1 | *Myotis species* | 305 (223-395) | 103 (63-172) | 13 | 11 |
| MyV2 | *Myotis species* | 261 (182-330) | 216 (154-288) | 20 | 25 |
| PhV | *Parastrellus hesperus* | 255 (182-335) | 205 (131-288) | 25 | 38 |
| PsV | *Perimyotis subflavus* | 83 (61-107) | 43 (31-60) | 17 | 30 |

Table S1. Host species, divergence time summaries and sampling information for rabies virus lineages. The first estimate of the time since the most recent common ancestor (“Stem branch TMRCA”) includes the stem branch leading to existing lineage diversity. The second estimate of the TMRCA refers to existing viral genetic diversity, not including the leading stem branch. “SAV” notation refers to lineages found in South American bat populations/species that contain a congeneric North American member in the dataset.
