# Supplementary material for: Rates of Viral Evolution Are Linked to Host Geography in Bat Rabies
Source: PLoS Pathog. 2012 May 17;8(5):e1002720. doi: 10.1371/journal.ppat.1002720 (PMC3355098; doi:10.1371/journal.ppat.1002720)
Supplement: Table S2 — Ecological traits of bat species and data sources. Mass independent values of basal metabolic rate (BMR) and torpid metabolic rate (TMR) were used. Rates that were calculated using data on body mass and metabolic rates (see Materials and Methods) are indicated by “a". Climatic regions abbreviated as follows: TR = tropical; ST = subtropical; TE = temperate. Representative species trait values are reported for viruses that circulate in several species of Myotis in the western United States. (DOC) [file ppat.1002720.s003.doc]

| **Lineage** | **Host species** | **COI Accession number** | **BMR** | **TMR** | **Long distance migrant** | **Colonial** | **Seasonal activity** | **Climatic region** | **Sources** |
| --- | --- | --- | --- | --- | --- | --- | --- | --- | --- |
| DrV | *Desmodus rotundus* | EU096711 | 2.777 | 0.078 ^a^ | N | Y | Y | TR | [1-3] |
| TbSAV | *Tadarida b. brasiliensis* | JF446884 | 2.663 | 0.337 | Y | Y | Y | TR | [2, 4-7] |
| TbV | *Tadarida brasiliensis mexicana; T. b. cynocephala* | GU723091 | 2.663 | 0.337 | Y | Y | Y | ST | [2, 4, 5, 8, 9] |
| NlV | *Nyctinomops laticaudatus* | EF080535 | 1.887 | 0.053 ^a^ | N | Y | Y | TR | [2, 10, 11] |
| EfSAV | *Eptesicus furinalis* | EU096740 | 2.872 ^a^ | 0.080 ^a^ | N | Y | Y | TR | [1, 12, 13] |
| EfV1a | *Eptesicus fuscus pallidus* | GU723061 | 4.576 | 0.145 | N | Y | N | ST | [2, 14, 15] |
| EfV1b | *Eptesicus fuscus bernardinus* | GU723077 | 4.576 | 0.145 | N | Y | N | TE | [2, 9, 14, 15] |
| EfV2 | *Eptesicus fuscus fuscus* | GU723032 | 4.576 | 0.145 | N | Y | N | TE | [2, 9, 14, 15] |
| EfV3 | *Eptesicus fuscus fuscus; E. f. bernardinus* | GU723030 | 4.576 | 0.145 | N | Y | N | TE | [2, 9, 14, 15] |
| LbV1 | *Lasiurus borealis* | GU723006 | 3.264 | 0.099 | Y | N | N | TE | [2, 9, 14, 15] |
| LbV2 | *Lasiurus borealis* | GU722999 | 3.264 | 0.099 | Y | N | N | TE | [2, 9, 14, 15] |
| LcV | *Lasiurus cinereus* | GU644886 | 2.226 | 0.062 ^a^ | Y | N | N | TE | [2, 9, 14] |
| LiV | *Lasiurus intermedius floridanus* | GU722925 | 2.266 | 0.063 ^a^ | N | Y | Y | ST | [2, 9, 14, 16-18] |
| LsV | *Lasiurus seminolus* | GU723011 | 2.927 | 0.082 ^a^ | N | N | N | ST | [2, 9, 14, 19, 20] |
| LxV | *Lasiurus xanthinus* | GU722938 | 2.845 ^a^ | 0.079 ^a^ | Y | N | Y | ST | [9, 21-24] |
| LnV | *Lasionycteris noctivagans* | GU722947 | 2.864 ^a^ | 0.115 | Y | Y | N | TE | [14, 25, 26] |
| MySAV | *Myotis nigricans* | EU096808 | 1.535 | 0.043 ^a^ | N | Y | Y | TR | [1, 2, 27] |
| MyV1 | *Myotis yumanensis* | GU723131 | 2.891 | 0.081^d^ | N | Y | N | ST | [2, 9, 14, 28] |
| MyV2 | *Myotis californicus* | GU723116 | 2.651 | 0.057 | N | Y | N | TE | [2, 8, 9, 14, 29] |
| PhV | *Parastrellus hesperus* | GU723161 | 3.572 ^a^ | 0.100 ^a^ | N | Y | N | ST | [2, 8, 9, 14, 30] |
| PsV | *Perimyotis subflavus* | GU723149 | 3.130 ^a^ | 0.087 ^a^ | N | Y | N | TE | [2, 9, 14, 31] |

**Table S2**

Table S2. Ecological traits of bat species and data sources. Mass independent values of basal metabolic rate (BMR) and torpid metabolic rate (TMR) were used. Rates that were calculated using available information on body mass and metabolic rates (see Materials and Methods) are indicated by “a”. Climatic regions abbreviated as follows: TR = tropical; ST = subtropical; TE = temperate. Representative species trait values are reported for viruses that circulate in several species of *Myotis* in the western United States.

**Table S2 References**

1. Borisenko AV, Lim BK, Ivanova NV, Hanner RH, Hebert PDN (2008) DNA barcoding in surveys of small mammal communities: a field study in Suriname. Molecular Ecology Resources 8: 471-479.

2. Jones KE, Bielby J, Cardillo M, Fritz SA, O'Dell J, et al. (2009) PanTHERIA: a species-level database of life history, ecology, and geography of extant and recently extinct mammals. Ecology 90: 2648-2648.

3. Greenhall AM, Joermann G, Schmidt U (1983) Desmodus rotundus. The American Society of Mammalogists. 1-6 p.

4. Wilkins KT (1989) Tadarida brasiliensis. Mammalian Species: 1-10.

5. Herreid C, Schmidt-Nielsen K (1966) Oxygen consumption, temperature, and water loss in bats from different environments. American Journal of Physiology--Legacy Content 211: 1108.

6. Clare EL, Lim BK, Fenton MB, Hebert PDN (2011) Neotropical Bats: Estimating Species Diversity with DNA Barcodes. Plos One 6: e22648.

7. Romano MC, Maidagan JI (1999) Behavior and demography in an urban colony of Tadarida brasiliensis (Chiroptera: Molossidae) in Rosario, Argentina. Revista de Biologia Tropical 47: 1121-1127.

8. Geluso K (2007) Winter activity of bats over water and along flyways in New Mexico. The Southwestern Naturalist 52: 482-492.

9. Streicker DG, Turmelle AS, Vonhof MJ, Kuzmin IV, McCracken GF, et al. (2010) Host phylogeny constrains cross-species emergence and establishment of rabies virus in bats. Science 329: 676-679.

10. Avila-Flores R, Flores-MartÌnez JJ, Ortega J (2002) Nyctinomops laticaudatus. Mammalian Species: 1-6.

11. Clare EL, Lim BK, Engstrom MD, Eger JL, Hebert PDN (2007) DNA barcoding of Neotropical bats: species identification and discovery within Guyana. Molecular Ecology Notes 7: 184-190.

12. Mies R, Kurta A, King DG (1996) Eptesicus furinalis. Mammalian Species: 1-7.

13. Myers P (1977) Patterns of reproduction of four species of vespertilionid bats in Paraguay: University of California Press.

14. Boyles JG, Dunbar MB, Whitaker J, J.O. (2006) Activity following arousal in winter in North American vespertilionid bats. Mammal Review 36: 267-280.

15. Dunbar MB, Brigham RM (2010) Thermoregulatory variation among populations of bats along a latitudinal gradient. Journal of Comparative Physiology B: Biochemical, Systemic, and Environmental Physiology: 1-9.

16. Webster WD, Jones Jr JK, Baker RJ (1980) Lasiurus intermedius. Mammalian Species: 1-3.

17. Humphrey SR (1975) Nursery roosts and community diversity of Nearctic bats. Journal of Mammalogy: 321-346.

18. Kunz TH (1982) Lasionycteris noctavagans. Mammalian Species 172: 1-5.

19. Hein CD, Castleberry SB, Miller KV (2008) Sex-specific summer roost-site selection by seminole bats in response to landscape-level forest management. Journal of Mammalogy 89: 964-972.

20. Wilkins KT (1987) Lasiurus seminolus. Mammalian Species 280: 1-5.

21. O'Farrell MJ, Williams JA, Lund B (2004) Western Yellow Bat (Lasiurus xanthinus) in Southern Nevada. The Southwestern Naturalist 49: 514-518.

22. Higginbotham JL, Dixon MT, Ammerman LK (2000) Yucca provides roost for Lasiurus xanthinus (Chiroptera: Vespertilionidae) in Texas. The Southwestern Naturalist 45: 338-340.

23. Carter TC, Menzel JM, Lacki M, Hayes J, Kurta A (2007) Behavior and day-roosting ecology of North American foliage-roosting bats. Bats in forests: conservation and management: 61-81.

24. Bisson IA, Safi K, Holland RA (2009) Evidence for repeated independent evolution of migration in the largest family of bats. Plos One 4: e7504.

25. Vonhof MJ, Barclay RMR (1996) Roost site selection and roosting ecology of forest dwelling bats in southern British Columbia. Canadian Journal Of Zoology-Revue Canadienne De Zoologie 74: 1797-1805.

26. Dunbar MB (2007) Thermal energetics of torpid silver-haired bats Lasionycteris noctivagans. Acta Theriologica 52: 65-68.

27. Wilson DE, LaVal RK (1974) Myotis nigricans. Mammalian Species: 1-3.

28. Dalquest WW (1947) Notes on the natural history of the bat, Myotis yumanensis, in California, with a description of a new race. American Midland Naturalist 38: 224-247.

29. Speakman JR, Thomas DW (2003) Physiological Ecology and Energetics of Bats. In: Kunz TH, Fenton MB, editors. Bat Ecology. Chicago: The University of Chicago Press. pp. 430-490.

30. Cross SP (1965) Roosting habits of Pipistrellus hesperus. Journal of Mammalogy: 270-279.

31. Fujita MS, Kunz TH (1984) Pipistrellus subflavus. Mammalian Species 228: 1-6.
