# Supplementary material for: Rates of Viral Evolution Are Linked to Host Geography in Bat Rabies
Source: PLoS Pathog. 2012 May 17;8(5):e1002720. doi: 10.1371/journal.ppat.1002720 (PMC3355098; doi:10.1371/journal.ppat.1002720)
Supplement: Table S3 — Phylogenetic signal of bat and virus traits in the bat phylogeny as measured by Blomberg's K and Pagel's λ. Values of Blomberg's K were estimated for continuous traits with significance tested by 5000 randomizations of trait values on the phylogenetic tree of bats from COI sequences (Figure S1). Maximum likelihood (ML) values of λ were estimated using the Geiger package of R with significance determined by likelihood ratio tests comparing models assuming the ML estimate of λ to models assuming no phylogenetic signal (λ = 0) with 1 degree of freedom. When only two categories of climatic zone were included (tropical and subtropical versus temperate) the ML value of λ was 1; however, this estimate was only marginally significantly better than the model assuming λ = 0 (P = 0.06). (DOC) [file ppat.1002720.s004.doc]

**Table S3**

|  | **Blomberg’s *K*** | | **Pagel’s *λ*** | |
| --- | --- | --- | --- | --- |
| **Trait** | ***K*** | ***P*** | ***λ*** | ***P*** |
| Basal metabolic rate | 0.9 | < 0.001 | 1 | < 0.00001 |
| Torpid metabolic rate | 1 | < 0.001 | 1 | < 0.00001 |
| Range of years sampled | 0.011 | 0.720 | 1.0e-7 | 1 |
| Number of sequences | 0.004 | 0.996 | 1.0e-7 | 1 |
| Climatic region | Not done | Not done | 5.0e-5 | 1 |
| Coloniality | Not done | Not done | 1 | 0.005 |
| Seasonal inactivity | Not done | Not done | 1 | 0.016 |
| Long-distance migration | Not done | Not done | 1 | 0.051 |

Table S3. Phylogenetic signal in bat and virus traits measured by Blomberg’s *K* and Pagel’s *λ*. Values of Blomberg’s *K* were estimated only for continuous traits with significance tested by 5000 randomizations of trait values on the phylogenetic tree of bats from COI sequences (Figure S1). Maximum likelihood (ML) values of *λ* were estimated using the *Geiger* package of R with significance determined by likelihood ratio tests comparing models assuming the ML estimate of *λ* to models assuming no phylogenetic signal (*λ* = 0) with 1 degree of freedom. When only two categories of climatic zone were included (tropical and subtropical versus temperate) the ML value of *λ* was 1; however, this estimate was only marginally significantly better than the model assuming *λ* = 0 (*P* = 0.06).
