# Supplementary material for: Rates of Viral Evolution Are Linked to Host Geography in Bat Rabies
Source: PLoS Pathog. 2012 May 17;8(5):e1002720. doi: 10.1371/journal.ppat.1002720 (PMC3355098; doi:10.1371/journal.ppat.1002720)
Supplement: Table S4 — The confidence set of generalized linear models examined to explain viral evolutionary rate. All models included a significant intercept term and had full model P values<0.0001. Abbreviated terms are defined as follows: BMR: mass-independent basal metabolic rate; TMR: mass-independent torpid metabolic rate; n: number of sequences per lineage; nyrs: range of years spanned per lineage. AIC weights (w) describe the relative likelihood for each model given the set of models considered. (DOC) [file ppat.1002720.s005.doc]

**Table S4**

| **Model** | **AICc** | **Δ AICc** | ***w*** | ***r^2^*** |
| --- | --- | --- | --- | --- |
| Climatic region | 40.297 | 0.000 | 0.370 | 0.66 |
| Climatic region + long-distance migration | 42.710 | 2.413 | 0.111 | 0.67 |
| Climatic region + nyrs | 42.823 | 2.526 | 0.105 | 0.67 |
| Climatic region + log(BMR) | 43.173 | 2.876 | 0.088 | 0.67 |
| Climatic region + seasonal inactivity | 43.185 | 2.888 | 0.087 | 0.67 |
| Climatic region + log(TMR) | 43.311 | 3.014 | 0.082 | 0.66 |
| Climatic region + log(n) | 43.379 | 3.082 | 0.079 | 0.66 |
| Climatic region + coloniality | 43.384 | 3.087 | 0.079 | 0.66 |

Table S4. The confidence set of generalized linear models examined to explain viral evolutionary rate. All models included a significant intercept term and had full model *P* values < 0.0001. Abbreviated terms are defined as follows: BMR: mass-independent basal metabolic rate; TMR: mass-independent torpid metabolic rate; n: number of sequences per lineage; nyrs: range of years spanned per lineage. AIC weights (*w*) describe the relative likelihood for each model given the set of models considered.
