# Supplementary material for: Rates of Viral Evolution Are Linked to Host Geography in Bat Rabies
Source: PLoS Pathog. 2012 May 17;8(5):e1002720. doi: 10.1371/journal.ppat.1002720 (PMC3355098; doi:10.1371/journal.ppat.1002720)
Supplement: Table S5 — Phylogenetic generalized least squares regression support for the confidence set of models found in the generalized linear model analysis (see Table S4 for explanations of terms). Pagel's λ was estimated using the rabies virus phylogeny (topology from Figure 2A) and observed trait data. (DOC) [file ppat.1002720.s006.doc]

**Table S5**

| **Model** | **AICc** | **Δ AICc** | ***w*** | **λ** |
| --- | --- | --- | --- | --- |
| Climatic region | 28.308 | 0.000 | 0.314 | 0 |
| Climatic region + long-distance migration | 30.283 | 1.975 | 0.117 | 0 |
| Climatic region + nyrs | 30.200 | 1.892 | 0.122 | 0 |
| Climatic region + log(BMR) | 30.347 | 2.039 | 0.113 | 0 |
| Climatic region + seasonal inactivity | 30.281 | 1.973 | 0.117 | 0 |
| Climatic region + log(TMR) | 31.233 | 2.925 | 0.073 | 0 |
| Climatic region + log(n) | 31.252 | 2.944 | 0.072 | 0 |
| Climatic region + coloniality | 31.252 | 2.944 | 0.072 | 0 |

Table S5. Phylogenetic generalized least squares regression support for the confidence set of models found in the generalized linear model analysis. The confidence set of models from the GLM analysis is shown. See Table S4 for explanations of terms. Pagel’s λ was estimated using the rabies virus phylogeny (topology from Figure 2A) and observed trait data.
