# Supplementary material for: Rates of Viral Evolution Are Linked to Host Geography in Bat Rabies
Source: PLoS Pathog. 2012 May 17;8(5):e1002720. doi: 10.1371/journal.ppat.1002720 (PMC3355098; doi:10.1371/journal.ppat.1002720)
Supplement: Table S6 — Evolutionary rate estimates by codon partition for independent lineage models. Numbers in parentheses are the 95% highest posterior density around the median rate. (DOC) [file ppat.1002720.s007.doc]

**Table S6**

| **RV lineage** | **CP_12_** | **CP_3_** | **CP_123_** |
| --- | --- | --- | --- |
| DrV | 1.8e-4 (9.8e-5,2.8e-4) | 1.7e-3 (9.3e-4,2.6e-3) | 1.0e-3 (5.6e-4,1.6e-3) |
| TbSAV | 1.1e-4 (7.7e-6,2.2e-4) | 1.5e-3 (9.8e-5,2.8e-3) | 5.6e-4 (3.8e-5,1.1e-3) |
| TbV | 1.3e-4 (8.3e-5,1.8e-4) | 7.3e-4 (4.8e-4,1.0e-3) | 3.3e-4 (2.1e-4,4.6e-4) |
| NlV | 8.1e-5 (2.4e-5,1.6e-4) | 9.4e-4 (2.7e-4,1.8e-3) | 5.5e-4 (1.6e-4,1.1e-3) |
| EfSAV | 7.1e-5 (1.6e-5,1.4e-4) | 5.8e-4 (1.3e-4,1.2e-3) | 2.4e-4 (5.4e-5,4.8e-4) |
| EfV1a | 2.9e-5 (9.0e-7,6.7e-5) | 6.5e-4 (2.0e-5,1.5e-3) | 3.5e-4 (1.1e-5,8.0e-4) |
| EfV1b | 9.3e-6 (9.0e-7,3.4e-5) | 8.8e-5 (8.5e-6,3.2e-4) | 3.6e-5 (3.5e-6,1.3e-4) |
| EfV2 | 5.9e-5 (2.2e-5,9.3e-5) | 4.9e-4 (1.9e-4,7.8e-4) | 1.7e-4 (6.4e-5,2.7e-4) |
| EfV3 | 8.5e-5 (4.7e-5,1.2e-4) | 5.8e-4 (3.2e-4,8.5e-4) | 2.5e-4 (1.4e-4,3.7e-4) |
| LbV1 | 1.9e-5 (1.6e-6,8.3e-5) | 1.5e-4 (1.3e-5,6.8e-4) | 6.4e-5 (5.5e-6,2.8e-4) |
| LbV2 | 5.8e-5 (4.7e-6,1.3e-4) | 5.1e-4 (4.2e-5,1.2e-3) | 3.1e-4 (2.5e-5,7.2e-4) |
| LcV | 2.2e-5 (6.7e-7,6.0e-5) | 1.2e-4 (3.5e-6,3.1e-4) | 8.2e-5 (2.4e-6,2.2e-4) |
| LiV | 2.2e-4 (7.1e-5,3.5e-4) | 2.1e-3 (6.8e-4,3.3e-3) | 8.4e-4 (2.8e-4,1.3e-3) |
| LsV | 5.9e-5 (2.0e-7,1.5e-4) | 1.1e-3 (3.6e-6,2.7e-3) | 3.9e-4 (1.3e-6,9.8e-4) |
| LxV | 2.6e-4 (2.4e-5,6.2e-4) | 1.6e-3 (1.5e-4,3.8e-3) | 7.0e-4 (6.5e-5,1.7e-3) |
| LnV | 1.3e-5 (2.6e-6,3.0e-5) | 1.2e-4 (2.4e-5,2.8e-4) | 4.8e-5 (9.7e-6,1.1e-4) |
| MSAV | 8.3e-5 (3.7e-08,5.7e-4) | 1.4e-3 (6.4e-7,9.9e-3) | 5.3e-4 (2.4e-7,3.7e-3) |
| MV1 | 1.5e-4 (7.3e-7,3.5e-4) | 1.3e-3 (6.4e-6,3.1e-3) | 5.2e-4 (2.6e-6,1.3e-3) |
| MV2 | 2.1e-5 (4.1e-6,4.7e-5) | 2.8e-4 (5.6e-5,6.4e-4) | 1.1e-4 (2.1e-5,2.4e-4) |
| PhV | 9.8e-5 (2.2e-5,2.0e-4) | 1.1e-3 (2.6e-4,2.3e-3) | 4.5e-4 (1.0e-4,8.9e-4) |
| PsV | 4.1e-5 (6.8e-7,7.8e-5) | 3.1e-4 (5.2e-6,6.0e-4) | 1.3e-4 (2.2e-6,2.5e-4) |

Table S6. Evolutionary rate estimates by codon partition for independent lineage models. Numbers in parentheses are the 95% highest posterior density around the median rate.
