# Supplementary material for: Rates of Viral Evolution Are Linked to Host Geography in Bat Rabies
Source: PLoS Pathog. 2012 May 17;8(5):e1002720. doi: 10.1371/journal.ppat.1002720 (PMC3355098; doi:10.1371/journal.ppat.1002720)
Supplement: Table S7 — Evolutionary rate estimates by codon partition from the hierarchical phylogenetic model. Numbers in parentheses are the 95% highest posterior density around the median rate and were calculated conditionally on the portion of the posterior distribution for which the significant effect (climatic region) was included in the model (i.e., βi|δEffect i = 1). (DOC) [file ppat.1002720.s008.doc]

Table S7

| **RV lineage** | **CP_12_** | **CP_3_** | **CP_123_** |
| --- | --- | --- | --- |
| DrV | 1.0e-4 (6.4e-5,1.5e-4) | 1.2e-3 (7.9e-4,1.5e-3) | 3.6e-4 (2.6e-4,5.1e-4) |
| TbSAV | 6.0e-5 (1.0e-6,2.0e-4) | 1.2e-3 (6.5e-4,1.8e-3) | 3.2e-4 (1.5e-4,5.2e-4) |
| TbV | 1.3e-4 (7.6e-5,1.9e-4) | 7.3e-4 (4.8e-4,1.1e-3) | 3.2e-4 (2.3e-4,4.2e-4) |
| NlV | 5.5e-5 (1.2e-5,1.2e-4) | 1.1e-3 (6.7e-4,1.6e-3) | 3.1e-4 (1.8e-4,4.4e-4) |
| EfSAV | 9.2e-5 (2.7e-5,1.8e-4) | 1.0e-3 (5.7e-4,1.4e-3) | 3.2e-4 (1.7e-4,4.5e-4) |
| EfV1a | 3.4e-5 (7.6e-6,7.3e-5) | 3.1e-4 (1.5e-4,5.2e-4) | 2.7e-4 (8.5e-5,4.3e-4) |
| EfV1b | 2.2e-5 (8.4e-6,3.9e-5) | 2.7e-4 (1.3e-4,4.1e-4) | 6.8e-5 (2.6e-5,1.1e-4) |
| EfV2 | 3.5e-5 (1.5e-5,6.7e-5) | 3.3e-4 (1.8e-4,5.3e-4) | 8.5e-5 (3.3e-5,1.8e-4) |
| EfV3 | 3.7e-5 (1.9e-5,5.8e-5) | 2.9e-4 (1.7e-4,4.2e-4) | 7.8e-5 (3.6e-5,1.3e-4) |
| LbV1 | 2.5e-5 (8.6e-6,5.1e-5) | 2.2e-4 (9.5e-5,3.8e-4) | 6.4e-5 (2.1e-5,1.1e-4) |
| LbV2 | 2.7e-5 (9.2e-6,5.6e-5) | 2.4e-4 (1.1e-4,4.6e-4) | 7.0e-5 (2.4e-5,1.4e-4) |
| LcV | 3.8e-5 (1.5e-5,6.9e-5) | 2.1e-4 (1.0e-4,3.6e-4) | 6.5e-5 (2.5e-5,1.1e-4) |
| LiV | 1.1e-4 (2.7e-5,2.5e-4) | 1.3e-3 (7.3e-4,2.4e-3) | 3.6e-4 (1.7e-4,7.4e-4) |
| LsV | 4.1e-5 (1.0e-6,1.3e-4) | 1.2e-3 (5.3e-4,2.1e-3) | 2.7e-4 (5.1e-5,4.6e-4) |
| LxV | 1.4e-4 (7.9e-6,4.3e-4) | 1.2e-3 (6.6e-4,2.3e-3) | 3.1e-4 (8.8e-5,5.6e-4) |
| LnV | 2.0e-5 (7.5e-6,3.7e-5) | 1.9e-4 (9.1e-5,3.0e-4) | 6.5e-5 (2.6e-5,1.1e-4) |
| MSAV | 6.4e-5 (2.4e-5,1.2e-4) | 1.1e-3 (5.9e-4,1.8e-3) | 3.2e-4 (1.4e-4,5.6e-4) |
| MV1 | 9.7e-5 (2.7e-5,2.0e-4) | 1.1e-3 (5.6e-4,1.7e-3) | 3.0e-4 (6.9e-5,4.8e-4) |
| MV2 | 1.8e-5 (8.0e-6,3.0e-5) | 2.9e-4 (1.6e-4,4.6e-4) | 7.4e-5 (3.3e-5,1.3e-4) |
| PhV | 7.0e-5 (3.1e-5,1.2e-4) | 1.1e-3 (5.5e-4,1.6e-3) | 3.0e-4 (1.0e-4,4.6e-4) |
| PsV | 3.2e-5 (9.5e-6,6.7e-5) | 3.0e-4 (1.8e-4,4.7e-4) | 7.8e-5 (3.3e-5,1.4e-4) |

Table S7. Evolutionary rate estimates by codon partition from the hierarchical phylogenetic model. Numbers in parentheses are the 95% highest posterior density around the median rate and were calculated conditionally on the portion of the posterior distribution for which the significant effect (climatic region) was included in the model (i.e., *β_i_* | *δ_Effect i_* = 1).
